# Chronic Cystitis in the Daughter; Fibroid Polypus of the Bladder in the Mother; Extirpation and Laceration of the Bladder; Suture; Recovery

**Published:** 1882-11

**Authors:** O. Stroinski

**Affiliations:** Chicago


### ARTICLE VI.

CHRONIC CYSTITIS IN THE DAUGHTER; FIBROID POLYPUS OF THE BLADDER IN THE MOTHER; EXTIRPATION; LACERATION OF THE BLADDER; SUTURE; RECOVERY. By O. STROINSKI, M.D., Chicago.

Mrs. L., a young woman twenty-one years of age, was married about three years ago. When pregnant, in the fourth month, she noticed pains in urinating, and some blood escaping after the passage of urine from the bladder. The pains became intense in the fifth month of pregnancy, but no remedy was of any avail. After confinement, the pains subsided entirely for six weeks, and then reappeared in the old manner. Now, sixteen months after the birth of the child, the pains are intolerable. The woman is a healthy-looking person of medium size ; all the organs of the upper body are in healthy condition, and the functions of the body are perfectly normal, except that of the bladder. The uterus is retroflected, and the walls of the bladder intensely painful in examination. There is chronic cystitis of the bladder, resulting from active hyperæmia of the organ, which latter is so often found in pregnant women with a retroflected uterus. Three injections into the bladder of a strong solution of nitrate of silver, 10 to 100, were made, the organ having been emptied previously by a male catheter, at intervals of four days, and the bladder was washed each time with lukewarm salt water. The pains have entirely disappeared, and there is no escape of blood. The speedy cure of the daughter induced the mother, a refined lady of forty-six years, and lately arrived from Europe, to consult me about her complaints. She has had the same troubles when pregnant with this daughter, her first child, and she has suffered more or less during the last twenty-five years, especially when pregnant. In the last six months the pains became labor-like, and they were of so painful a character that she hid herself in some remote place, that her moans might not be heard by the family. The woman is a little taller and stouter than her daughter, but she told me that she had lost a good deal of flesh in the last year. The uterus is retroflected, as in the daughter. The walls of the bladder are also painful, as in the daughter, but the sound strikes against a foreign body on the anterior wall of the bladder. This body is movable, but it will be found always in the same place when attacked from one or the other side. I rapidly dilated the urethra with the index-finger, and the finger also detected a moveable body. That this foreign body was not a stone in the bladder the symptoms would readily show, and the introduced sound could not feel a hard body giving the characteristic clink of the stone, but a soft body moving from one side to the other, but being attached with its basis to the bladder. The diagnosis was therefore polypus of the bladder. The mucous membrane of the bladder could be easily displaced from its muscular layer in some parts, a circumstance which aided me a good deal in the coming operation. With the assistance of Dr. Patzer, a surgeon lately arrived from Russia, I chloroformed the patient, and then dilated the urethra as before. A Simon's curved forceps was introduced, and after several attacks the short and dense pedicle of the growth was seized within its blades. About ten rotations were necessary to remove the growth, which was of the size of a walnut, and of dense fibrous structure. I then injected a moderate quantity of lukewarm salt water, but which could not be withdrawn. The woman suddenly collapsed, and on palpation of the abdominal walls, there could be detected a quantity of fluid in the abdominal cavity. The bladder had ruptured; or, to use the proper technical term, it was lacerated by the tractions on the fibroid. By pressure on the abdomen with a tight closing syringe in the bladder, I was able to remove a large quantity of the fluid. The woman rallied. I introduced a permanent catheter, and left the woman in comfortable circumstances. On account of the rare occurrence of spontaneous healing in a ruptured bladder, and in consideration of the age of the woman and the peculiar condition of the walls of the bladder, I thought it necessary to suture the bladder, and to effect thus a closure of the rent. Through the vagina I could not operate, the laceration being on the anterior part of the bladder, and I was therefore compelled to make a complete inversion of the organ through the urethra. The mucous membrane near the sphincter, which could be easily displaced, I inverted first, and I laid two ligatures through this part. By pulling on these ligatures, and assisted by Dr. Patzer, who made a strong pressure above the symphysis upon the bladder, I effected a complete inversion. The mucous membrane was in a state of chronic inflammation, and the walls around the removed tumor were exceedingly thin. Here was a rent two cm. long, and in diagonal direction. Three carbolized silk sutures sufficed to close the aperture. The reduction of the bladder was easily effected by a repositor. A lukewarm injection of salt water was made, and the liquid did not enter the abdominal cavity. A permanent catheter was kept *in situ* for two weeks. The woman has now entirely recovered. She has no pains in urinating, and there is no show of blood. The retroflexion was treated with the usual pessary. Fibroma of the bladder is a rare occurrence, and it will be found only after long-continued inflammation.

